# Anaesthetizing children—From a nurse anaesthetist's perspective—A qualitative study

**DOI:** 10.1002/nop2.147

**Published:** 2018-04-20

**Authors:** Lena Danielsson, Marie‐Louise Lundström, Inger K. Holmström, Birgitta Kerstis

**Affiliations:** ^1^ Region Västmanland Västmanlands sjukhus Västerås Sweden; ^2^ School of Health, Care and Social Welfare Mälardalen University Västerås Sweden; ^3^ Department of Public Health and Caring Sciences Uppsala University Uppsala Sweden

**Keywords:** anaesthetics, children, nurses, nursing, parents

## Abstract

**Aim:**

The aim of this study was to describe nurse anaesthetists' experiences of encountering and caring for children in connection to anaesthesia.

**Design:**

Qualitative design.

**Methods:**

Sixteen written narratives based on eight nurse anaesthetists' experiences of meeting children was analysed using qualitative content analysis.

**Results:**

The overarching theme was: “anaesthetizing children is a complex caring situation, including interactions with the child and parents as well as ensuring patient safety, affected by the perioperative team and organizational prerequisites”. The nurses stated that in their interaction with the family, their goal was to ensure that children and parents felt secure and calm. “Striving to work in confidence” underlined the team and organizational influences. Encountering children involves more than knowledge about technical equipment, procedures and drugs. Knowledge about children's development and fears and parents' needs are essential for an optimal caring situation. Organizations need to realize that extra time, skills and resources are needed to safely anaesthetize children.

## 1 INTRODUCTION

### 1.1 Background

In Sweden, nurse anaesthetists are registered nurses with a 1‐year speciality education in anaesthetic nursing. They work rather independently, in contrast to many countries, where the nurse anaesthetist's role is more to assist the physician (Larsson & Holmström, 2013). In Sweden there were 10,550 nurse anaesthetists registered 2014 (Socialstyrelsen 2014). Nurse anaesthetists meet patients of different ages in their professional practice. A particularly vulnerable group of patients to anaesthetize and care for are children. Anaesthetizing children might push nurse anaesthetists' professional competence to the edge, both technically and emotionally. Factors which can cause stress for nurse anaesthetists when caring for children include intubation difficulties, staff shortages and/or high workload (Perry, 2005). It is important that nurse anaesthetists have knowledge of children's anatomy, breathing, circulation and pharmacokinetics, as these are different compared with adults (Clarke, 2010).

It is also every child's right to be listened to and get correct information (UNICEF, 1989) when in need of health care. This can present a dilemma, for instance in situations where a child needs to be anaesthetized. It important that nurse anaesthetists instil confidence and trust in children and their parents (Lindwall & von Post, 2009), as children can be afraid of unknown situations. Supporting children during medical procedures, such as anaesthesia, should be characterized by an effort to reach the child's horizon of understanding (Karlsson, Rydström, Enskär, & Dalheim Englund, 2014). Health professionals should be honest and allow children to respond and act in compliance with their concern or refusal to cooperate (Månsson & Dykes, 2004). However, ethical conflicts may arise when children refuse to cooperate; therefore, it is important that nurse anaesthetists reflect on ethical dilemmas and alternative solutions (Runeson, Proczkowska‐Björklund, & Idvall, 2010). Children's own decision‐making should be considered, although painful procedures are sometimes necessary (Månsson & Enskär, 2008; Runeson et al., 2010). Furthermore, flexibility in action is important to avoid physically restraining children (Berglund, Ericsson, Proczkowska‐Björklund, & Fridlund, 2013). For continuity, it is optimal if the nurse anaesthetists performing the anaesthesia meet the child both pre‐ and postoperatively (Lindwall & von Post, 2009). A Swedish study describes that children who participated well in the premedication procedure were less afraid and reluctant to cooperate during the remaining procedures (Proczkowska‐Björklund, Runeson, Gustafsson, & Svedin, 2008).

Nurse anaesthetists also need to be aware of the importance of supporting children and parents in their caregiving activities to increase their satisfaction with healthcare services (Sigurdardottir, Garwick, & Svavarsdottir, 2017). Key strategies for avoiding physical restraint include being sensitive to the child and being flexible in altering one's actions (Berglund et al., 2013). An anxious child increases the risk of postoperative pain and future sleep problems (Kain, Mayes, Caldwell‐Andrews, Karas, & McClain, 2006) and they can be more challenging to anaesthetise. According to the UN Convention on the Rights of the Child, every child should be met with respect, understanding and equality and have the same rights as adults (UNICEF 1989). The best interests of the child should always be at the forefront, meaning that all healthcare professionals should have knowledge of children's rights. Since 2015, Sweden has a new patient law stating that children should be specifically considered in healthcare and that all healthcare professionals should consider what is best for the individual child (Sveriges Kommuner och Landsting 2015). This also holds for nurse anaesthetists' professional practice. There is a need to describe nurse anaesthetists' experiences of encountering and caring for children in connection to anaesthesia. In the absence of such knowledge, the foundation for development of intervention strategies to address deficiencies will remain problematic.

### 1.2 Aim

Caring for children can hence be a challenge for experienced nurse anaesthetists as well. It is therefore important to highlight this group's experiences of caring for children and to learn from them to ensure that children receive optimal care when receiving anaesthesia. Therefore, the aim of this study was to describe nurse anaesthetists' experiences of encountering and caring for children in connection to anaesthesia.

## 2 THE STUDY

### 2.1 Design

A descriptive design with a qualitative content analysis of reports written by nurse anaesthetists about their experiences was used.

### 2.2 Sample and setting

A purposeful sample of nurse anaesthetists from different clinics in a county council in mid‐Sweden was chosen. We strived to include both male and female nurse anaesthetists, with both extensive and shorter experiences of anaesthetizing children. Inclusion criteria were having a specialist education as a nurse anaesthetist and having experience of anaesthetizing children. Ten nurse anaesthetists (four men and six women, with professional experience between 6 and 25 years), hereafter also referred to as nurses, were invited to participate and all agreed. The nurses received instructions and an anonymous postage‐paid envelope. They were also asked to provide their mobile phone number and after a week a reminder was sent to all mobile phones. Two additional reminders were subsequently sent. Ultimately, eight nurses participated in the study.

### 2.3 Data collection

Critical Incident Technique (CIT) was used for the data collection. This is a qualitative approach and is considered suitable when the aim is to find solutions for mainly practical problems. Flanagan (1954) is regarded as the founder of the method, which has subsequently been used more frequently in the healthcare sciences to capture patients' views and perceptions of care (Flanagan, 1954). According to Kemppainen (2000), the goal of CIT is to help the participant describe specific situations with as many relevant details as possible (Kemppainen, 2000). Respondents describe a situation from the criteria posed, aspects important for this situation and how it affected them. The researcher then searches the material for critical situations relevant to the phenomenon and the problem to be solved.

In this study, data were collected by means of written narratives about the nurse anaesthetists' experiences of encountering children in connection to anaesthesia. The participants were provided with written instructions to give two narrative descriptions each about one situation they perceived as a good experience of anaesthetizing a child and one situation they perceived as a less good situation. The nurse anaesthetists' also received nine questions. The questions concerned theirs′ working experiences, age of the children in question, their experiences when taking care of the children, the interaction with the children, parents and colleagues and conditions for taking care of children at the ward. In total, we received 16 written narratives. A pilot test was conducted, and some minor adjustments were made before collecting the nurses' narratives.

### 2.4 Data analysis

The written narratives were analysed using qualitative content analysis with an inductive approach (Flanagan, 1954), which strives to uncover common features and patterns in a text and generate a theoretical description of the phenomenon in focus (Graneheim & Lundman, 2004).

The analysis process followed the steps outlined by Graneheim and Lundman (2004). In the first step, authors LD, MLL and IKH read the narratives several times to get a grasp of the whole. In the second step, meaning units were identified in the text, which were condensed in the third step. The condensed text was then sorted into categories and subsequently sub‐themes. The analysis was then revised by the fourth author and some alterations were made. Disagreements were settled through negotiated consensus. As the present content analysis was latent, which means that an interpretation was made in the final step of the analysis, a theme was established. Hence, underlying meanings could be made visible. Examples of the steps in the analysis are provided in Table 1.

**Table 1:** Example of steps in the analysis

Meaning unit	Condensed meaning unit	Code	Category	Sub‐theme
The mother's calm attitude made me relax and feel calm too. This made it possible for me to approach the child in a natural way, without effort, to instil calmness. The mother in this case is an important aspect of my work.	A calm parent calms down both nurse and child.	A calm parent instils calmness in the caring situation.	Interaction with the parents	Interaction with the family

### 2.5 Ethics

The study follows the Swedish law for ethical vetting (2003:460) and was approved by the local university board. The heads of the departments where the nurse anaesthetists worked were first informed and then gave permission for the study to be conducted. Thereafter, the nurses were informed both orally and in writing about the study procedures and aims and of their right to withdraw from the study at any stage without having to provide an explanation. They then gave their informed consent to participate. Data were treated confidentially, with no identification of which nurse each quote is related to, to uphold the confidentiality criterion. The study was performed according to the principles established by the Declaration of Helsinki (World Medical Association 2013).

## 3 RESULTS

### 3.1 Overview of major findings

A theme was established—*Anaesthetizing children is a complex caring situation, including interacting with the child and the parents as well as ensuring patient safety and is affected by the perioperative team and the organizational prerequisites*—which appeared prominently in all narratives. This was built up by two sub‐themes and four categories describing nurse anaesthetists' experiences of encountering children in connection to anaesthesia (Table 2). The nurses stated that their goal was to create interactions to ensure that the children and parents felt secure and calm. Striving to work in confidence underlined the impact of the team and the organizational influences. The sub‐themes and categories are described below, illustrated with quotes.

**Table 2:** Categories, sub‐themes and theme describing nurse anaesthetists' experiences of encountering and caring for children in connection to anaesthesia

Categories	Sub‐themes	Theme
Interaction with the child Interaction with the parents	Interaction with the family	Anaesthetizing children is a complex caring situation, including interacting with the child and the parents as well as ensuring patient safety and is affected by the perioperative team and the organizational prerequisites.
Team influences Organizational influences	Striving to work in confidence	

### 3.2 Interaction with the family

The first sub‐theme includes two categories describing the complexity of having to care for and interact with two parties at the same time: *Interaction with the child* and *Interaction with the parents*.

#### 3.2.1 Interaction with the child

The nurses emphasized the importance of the immediate establishment of a well‐functioning contact with the child. They stated that they turned directly to the child and tried to make them feel like the most important person in the room. The nurses tried to praise and empower the child, as something out of the ordinary was about to happen. To arouse the child's curiosity, they asked them questions and showed them the equipment in the operating theatre:

…it's a great challenge to anaesthetize children. You have a few seconds to establish contact and that's when you say hello. If you at that moment manage to make eye contact with the child and the child answers promptly, well then the chances increase that everything will work out well. (Nurse 3)

The nurses underlined the importance of involving the child in that about to happen. They stated that they encouraged the child to speak and tell their own story. Difficulty in the communication could arise when a family was non‐native Swedish speakers, or when the child had some kind of disability, which could hinder the establishment of good contact.

An ambition to encounter the child respectfully was clear in the narratives. The nurses stated that they based their actions on previous experiences of anaesthetizing children. They listened to not only the child's request, but also had to stick to the plan outlined beforehand:

My thoughts are that I want to encounter the child with respect and integrity, as far as possible. I don't know about any evidence regarding how to accomplish this in reality. I just go on my gut feeling. (Nurse 3)

#### 3.2.2 Interaction with the parents

The nurses described that the parents' roles were crucial for the outcome of anaesthetizing children. When the present parent was calm and safe, the contact with the child often worked out well:

The father and the boy are settled right away, and the interaction works well. He sits near the boy's head and holds his hand. The boy's in pain, but he's calm. We don't interfere with their interaction, but let them have this moment to themselves. (Nurse 5)

The younger the child, the more important the parental involvement. An important aspect was that the parent should be well informed about that about to happen, which was underscored in the nurses' narratives. Despite the nurse anaesthetists' professional competence, the support from the accompanying parent was the most pertinent aspect for establishing good contact with the child:

This encounter affected me by offering further evidence of the importance of parental involvement. Despite our competence, we cannot persuade a child to cooperate if the parent is not on board and every healthcare interaction has a huge effect on future healthcare encounters. (Nurse 1)

The nurses described that there was generally not enough time to inform the accompanying parent about what was going to happen. Sometimes they perceived that the information was provided at the wrong time and place:

I do believe that this information was provided at the wrong moment. Just before we were about to put her to sleep, the anaesthesiologist and I informed them about what we were planning to do. One might question how receptive the child and the mum were at this particular moment.(Nurse 2)

The nurses also described situations that had worked out less well due to insufficient interaction with the parent. As one nurse described:

…this caring situation turned out a bit awkward. I felt that my contact with the child was well functioning. He was calm and I was calm, but the dad was furious. Because of that, I felt frustrated on behalf of the child'.(Nurse 4)

In the cases when the parent was scared, stressed and felt unsafe, the cooperation between nurse and child was often hampered. The nurses emphasized that they really tried to have the parents cooperate with them so that the anaesthesia would go smoothly and have as little negative effect as possible on the child and the parents.

### 3.3 Striving to work in confidence

Striving to work in confidence includes two categories describing how to enable safe and well‐functioning anaesthesia for children: *Team influences* and *Organizational influences*. These categories are closely related, as the preconditions for teamwork are dependent on organization, leadership, time allocation and financial preconditions.

#### 3.3.1 Team influences

The nurses described how important well‐functioning interaction with other professions was when anaesthetizing children. They expressed that each person on the team had a central role and that it was important for all of them to have knowledge about the anaesthesia procedure chosen for the child. According to the nurses, the person on the team who had the best contact with the child, regardless of profession, should be in charge of the contact during the whole encounter. At the same time, it was deemed crucial to keep the number of persons involved in the care and anaesthesia as low as possible, to avoid worrying the child:

Then we went into the operating theatre, only the three of us, together with a colleague of mine who stood at the back of the room. I think the latter is important, since yet another person in the discussion might stress or scare the child. (Nurse 1)

Teamwork was especially important when something unexpected occurred. For instance, deviances in the communication between the nurse anaesthetists and the anaesthesiologist created uncertainty and affected teamwork negatively. When the teamwork was functioning well and all present staff had clear roles, they often communicated merely by eye contact. Sometimes children were anaesthetized at satellite clinics and in such situations, the nurses felt extra vulnerable as their colleagues were far away:

When we work at so‐called satellite clinics, there are no colleagues to help if something happens. (Nurse 7)

#### 3.3.2 Organizational influences

The nurses emphasized the importance of preparation and of having a plan when encountering the child and the accompanying parent, to ensure patient safety. They described that non‐optimal situations were often caused by not having the time to read the patient's record, prepare the drugs, or check all the necessary equipment. A lack of planning time before meeting the child and the parents could be stressful for the nurses:

When I anaesthetize children, I want to have control over reasonable medication doses and know the plan ahead of time, to make it as good an experience as possible for both child and parent. …I was checking the intubation equipment and medications in the operating theatre when the anaesthesiologist entered with the child and parent. I had not had the time to go through the patient's record or check the doses and we had not discussed how we were going to anaesthetize. I felt annoyed and stressed. (Nurse 6)

It was commonly expressed in the narratives that there was a lack of support from managers in connection to anaesthetizing children. The nurses described that when surgery programmes were made, the fact that the patient was a child was not considered; it was planned like any other surgery, allowing no time for unpredictable matters or for preparation:

I consider the production to be the focus in my department. There's no time to do that little extra something. I feel that the managers don't understand that it's not the same thing to anaesthetize a child compared to an adult.(Nurse 6)

The nurses also highlighted the need for education about paediatric care, partly for themselves but also for their managers. They expressed that most of their knowledge about anaesthetising children was built on experience and tacit knowledge and less on scientific evidence. They described that they lacked opportunities to improve their competence in caring for children in different situations. In addition, the nurse anaesthetists described lacking support from managers regarding the responsibility involved in caring for and anaesthetizing children. Even if their colleagues supported them and affirmed their competence, they sometimes felt inadequate and that they not had given the child the best care possible.

Furthermore, the nurses underscored the importance of collaboration with hospital clinics involved in the child's care pre‐ and postsurgery. For instance, they wanted the child to have an intravenous catheter and premedication before coming to their department. If the child did not have an intravenous catheter this could cause the nurse anaesthetist stress, especially if the child was worried:

Without having any evidence, I think it's good if the child gets the intravenous catheter at their home clinic.(Nurse 3)

## 4 DISCUSSION

The aim of this study was to describe nurse anaesthetists' experiences of encountering and caring for children in connection to anaesthesia. The main finding is that anaesthetizing children is a complex caring situation, including interacting with the child and the parents as well as ensuring patient safety and is affected by the perioperative team and the organizational prerequisites.

One of the nurse anaesthetist's tasks is to create trust with the children and parents so that they feel safe (Lindwall & von Post, 2009). The nurses also emphasized the challenges of encountering and caring for children in connection to anaesthesia, including their demands for high competence and knowledge, so the child and the parents can be confident and secure and so they themselves can work evidence‐based and feel satisfied with their work. How the children are handled can have short‐ and long‐term consequences. In the short term, problems in connection to anaesthesia in children can cause adverse organizational outcomes, such as delayed surgery for the child and other patients and even more important, anxiety and behavioural reactions from the child (Hägglöf, 1999). In the long term, problems in connection to anaesthesia in children can increase the risk that they will have sleep problems in the future (Kain et al., 2006) and create problems for them in future encounters with health care (Finnström, Käck, & Söderhamn,^2011^).

Trust can be instilled through conversation and being sensitive (Karlsson et al., 2014). The nurse can also use the child's imagination and play with them to enhance their adaption to the environment and the situation (Månsson & Enskär, 2008). This demands knowledge about children of different ages, involving not only physiology but also cognitive and psychological development. The present nurses emphasized the importance of good interaction with the child and parents. They wanted the child to tell their story and to show the child and the parents the hospital equipment and premises. The nurses described that when the parents were calm and safe, the contact with the child often worked well. When nurses use humour or talk to a child about other things, this can reduce the child's concern (Chorney et al., 2009). It is important that professionals have knowledge about words that may trigger the children's negative experiences (Perry, Samuelsson, & Cyna, 2015). The nurses claimed that it was good for the children and their parents to meet the nurse afterwards. In often‐slimmed hospital organizations, however, this might be a difficult goal to achieve. Different strategies could help the communication with the child and parents, as a recent study describes that children receiving web‐based interactive preoperative information were better informed than those who received conventional print material (Lööf, Liljeberg, Eksborg, & Lönnqvist, 2017). Meeting the child and the parents afterwards might be even more important if the anaesthesia included moments of loss of control for the child, for example, involving physical restraint. The postoperative meeting can be an opportunity for reflection and for handling negative feelings that might occur in connection to anaesthesia, which can be important for the children in future situations (Lindwall & von Post, 2009). In addition, nurse anaesthetists can learn and develop their practice from these postoperative encounters. The nurses described dilemmas such as the child not cooperating, when holding them might seem like the only solution. It is important that nurses regularly reflect on ethical dilemmas that can arise when caring for children in connection to anaesthesia and discuss alternative solutions (Runeson et al., 2010).

The nurses sometimes had non‐optimal working conditions and possibilities, for instance not having the time to read a patient's record or prepare the drugs or equipment needed, which they feared could decrease patient safety. They also described that there was no extra time planned for children compared with when adults were to be given anaesthesia, which sometimes led to problems, as there are differences in handling children compared with adults (Clarke, 2010). According to Donabedian's model for evaluating the quality of health care, quality of care is related to three aspects: structure, process and outcomes (Donabedian, 1988) of care. In the context of child anaesthesia, this means that it is not only the competence of the nurses and the team that needs to be of a high standard for providing safe care. In addition, the structures—in the form of organizstional prerequisites—need to take into account the extra time, skills and education needed to anaesthetize children. One way to do this is to develop preparation programmes and implement them systematically (Holaday & Bar‐Mor, 1997). The teams also need time to debrief and to train in administering child anaesthesia in safe surroundings such as simulations (Green, Tariq, & Green, 2016), to ensure safe and optimal care.

### 4.1 Limitations and strengths

These results should be interpreted in the light of several limitations. First, the study used data were collected by means of written narratives about the nurse anaesthetists' experiences of encountering children in connection to anaesthesia. Just because a lot has been written does not mean that all areas have been included. It might have been an advantage of using an interview instead which would have made it possible to repeat a question for deepening an answer if required. However, an advantage of using written narratives is difficulty getting to multiple sites. Further had we no problems with legibility of the writing since the participants typed their responses. The study sample of eight nurse anaesthetists was rather small, but the 16 narratives provided rich data with thick descriptions and sufficient information power (Malterud, Siersma, & Guassora, 2015).

A qualitative inductive approach was used to describe nurse anaesthetists' experiences of encountering children. The trustworthiness of the results was ensured by thoroughly describing participants, data collection procedures and the analysis steps. Credibility was attained using a semi‐structured instruction guide and, after a pilot, some minor changes were made. The pilot‐tested narratives were not used in this study. The first, second and third authors compared the steps in the analysis and all authors discussed the emerging themes and categories. The credibility was enhanced as robustness was found when the nurses independently stated similar findings. Dependability was achieved as the narratives were independently read and analysed by researchers with backgrounds as nurse anaesthetists and/or paediatric nurses. The Swedish context should be considered with regard to transferability.

## 5 CONCLUSION

The anaesthesia nurse's profession includes more than knowledge about technical equipment and drugs; caring and knowledge, about children's development and fears is essential for an optimal caring situation. The nurses should also satisfy the parents' needs, something that is often necessary for the child's confidence. The nurses described that a well‐functioning team was necessary for the safe care of the child. Taking joint responsibility for a child imposes higher demands on cooperation between the professionals involved.

## 6 CLINICAL IMPLICATIONS

Working towards a common goal with good communication within the team is important for the nurses' working conditions, as well as for the child's and parents' confidence and safety. A child who falls asleep calmly in the context of anaesthesia will most likely wake up just as calmly. The organization needs to account for that extra time, skills and resources are needed to safely anaesthetize children.

## CONFLICT OF INTEREST

The authors declare that there are no conflicts of interest.

## AUTHOR CONTRIBUTIONS

LD and ML: contributed to the data collection, analysis and drafting of the manuscript. IH: contributed to the study conception and design, data collection, analysis and drafting of the manuscript. BK: contributed to analysis and drafting of the manuscript.
